# Sigmoid volvulus: outcomes of treatment and predictors of morbidity and mortality

**DOI:** 10.1007/s00423-022-02428-5

**Published:** 2022-01-14

**Authors:** David Moro-Valdezate, José Martín-Arévalo, Vicente Pla-Martí, Stephanie García-Botello, Ana Izquierdo-Moreno, Leticia Pérez-Santiago, Jorge Manuel Pedrós-Giménez, Rosana Villagrasa, Andrés Peña, Alejandro Espí-Macías

**Affiliations:** 1 Colorectal Surgery Unit, Department of General and Digestive Surgery, Biomedical Research Institute INCLIVA, Hospital Clínico Universitario de Valencia, Av. Blasco Ibáñez, 17. 46010 Valencia, Spain; 2 Department of Surgery, University of Valencia, Valencia, Spain; 3 Digestive Disease Department, Endoscopy Unit, Biomedical Research Institute INCLIVA, Hospital Clínico Universitario de Valencia, Valencia, Spain

**Keywords:** Intestinal volvulus, Colorectal surgery, Risk factors, Prognosis, Survival

## Abstract

**Purpose:**

To analyze the treatment outcomes for sigmoid volvulus (SV) and identify risk factors of complications and mortality.

**Methods:**

Observational study of all consecutive adult patients diagnosed with SV who were admitted from January 2000 to December 2020 in a tertiary university institution for conservative management, urgent or elective surgery. Primary outcomes were 30-day postoperative morbidity, mortality and 2-year overall survival (OS), including analysis of risk factors for postoperative morbidity or mortality and prognostic factors for 2-year OS.

**Results:**

A total of 92 patients were included. Conservative management was performed in 43 cases (46.7%), 27 patients (29.4%) underwent emergent surgery and 22 (23.9%) were scheduled for elective surgery. Successful decompression was achieved in 87.8% of cases, but the recurrence rate was 47.2%. Mortality rates following episodes were higher for conservative treatment than for urgent or elective surgery (37.2%, 22.2%, 9.1%, respectively; *p* = 0.044). ASA score > III was an independent risk factor for complications (OR = 5.570, 95% CI = 1.740–17.829, *p* < 0.001) and mortality (OR = 6.139, 95% CI = 2.629–14.335, *p* < 0.001) in the 30 days after admission. Patients who underwent elective surgery showed higher 2-year OS than those with conservative treatment (*p* = 0.011). Elective surgery (HR = 2.604, 95% CI = 1.185–5.714, *p* = 0.017) and ASA score > III (HR = 0.351, 95% CI = 0.192–0.641, *p* = 0.001) were independent prognostic factors for 2-year OS.

**Conclusion:**

Successful endoscopic decompression can be achieved in most SV patients, but with the drawbacks of high recurrence, morbidity and mortality rates. Concurrent severe comorbidities and conservative treatment were independent prognostic factors for morbidity and survival in SV.

## Introduction

Colonic volvulus is the third leading cause of large bowel obstruction worldwide. However, its infrequent incidence in western countries has led to a lack of experience in managing this event [1, 2].

Most studies include young patients from endemic areas, with low morbi-mortality rates, but with disparities in treatment. Nevertheless, in occidental countries, sigmoid volvulus (SV) usually appears in elderly patients with several other disorders, so high complication rate is associated. Adequate management plays a crucial role in patient clinical course [3–5].

In patients presenting with SV without peritonitis or colonic gangrene, the recommended treatment is the endoscopic detorsion in the acute setting, with subsequent elective surgery due to the high recurrence (43–75%) and mortality rates (15–40%) associated with conservative treatment alone [6–8]. However, there is a subset of frail patients with high surgical risk that could be managed conservatively. Decision-making as to which patients should undergo elective surgery or conservative treatment remains controversial due to a lack of definitive selection criteria and risk factors.

The endpoint of this study was to analyse outcomes in the different SV treatment options and identify independent risk factors of morbidity and mortality after treatment at a single centre in a non-endemic country.

## Material and methods

### Study design and setting

An observational study was conducted including consecutive adult patients diagnosed with SV from January 2000 to December 2020 in a tertiary university institution, University Clinic Hospital of Valencia, Spain.

### Patients

The study included all patients diagnosed with SV, admitted to the emergency department who underwent conservative management with endoscopic decompression, urgent surgery or elective surgery. No exclusion criteria were applied.

### Management of colonic volvulus

The first-line treatment for SV was endoscopic decompression. However, urgent surgery was performed in patients with colonic gangrene, peritonitis or when endoscopic treatment was unsuccessful. After endoscopic detorsion, the decompression tube was left in place for 1–3 days, following which elective surgery was usually scheduled. Nevertheless, conservative treatment alone was decided individually in frail patients with high surgical risk contraindicating surgery or when the patient or the family refused surgery. Surgical procedures were stratified into resection procedures (including sigmoid colectomy and subtotal colectomy) with or without anastomosis, and non-resection procedures with diverting stoma. The decision to perform anastomosis was taken according to operative findings, the systemic condition of each patient and the experience of the operating surgeon.

### Data collection and study variables

Patient data were acquired from hospital and primary care clinical records. Patient variables were age, sex, American Society of Anaesthesiologists (ASA) score, Charlson index and comorbidities (neurological disorder, hypertension, myocardial infarction, chronic heart failure, diabetes mellitus, chronic pulmonary disease, chronic renal failure, dementia, bedridden, previous neoplasm, chronic constipation and previous abdominal surgery). Patients with severe comorbidities were those with ASA score over III and Charlson index higher than 6 [9]. Variables related to clinical presentation were abdominal pain, obstipation, abdominal distension, gangrene, septic shock and digestive bleeding. The diagnosis tests recorded were plain abdominal X-ray and CT-scan. Management variables were number of episodes, endoscopic decompression, total of endoscopic decompressions, surgical or conservative management, type of surgery (urgent or elective), surgical procedure (resection with anastomosis, resection without anastomosis, diverting stoma), reoperation, length of hospital stay and readmission. Recurrence was any new volvulus occurring after endoscopic or surgical treatment.

### Outcomes

The primary outcomes were complications and mortality during the 30 days after admission and 2-year overall survival (OS), including analysis of possible risk factors for complications or mortality and prognostic factors for 2-year OS. Secondary outcomes were medical complications, surgical complications, surgical site infection (superficial, deep or organ space), cause of death and Clavien-Dindo classification (severe complications were those with a score higher than 2).

### Ethics

The study was approved by the local Research Ethics Committee.

### Statistical analysis

A descriptive study of the sample was carried out by analysing the characteristics of each variable. The normality of the variables was determined by the Shapiro–Wilk test. Qualitative variables were expressed as median and range. Fisher’s exact test or x^2^ test was used to find possible differences between qualitative variables while Mann–Whitney test was used for quantitative variables. In order to identify a possible pooling pattern of the treatment modalities according to time, a cluster analysis (a type of unsupervised learning technique) was carried out and the optimal number of clusters was calculated on the basis of the highest inter-cluster inertia. Furthermore, a logistic binary regression was made to identify independent risk factors for postoperative complications or mortality. Two-year overall survival (OS) was analysed by Kaplan–Meier method with between-group differences compared by log-rank test. Cox regression was conducted to estimate hazard ratios, indicating the effects of prognostic factors that could modify survival. *P* value < 0.05 was considered statistically significant. Statistical analysis was performed using IBM SPSS Statistics for Macintosh, version 25 (IBM Corp., Armonk, NY, USA).

## Results

### Patients’ features

The study included 92 patients diagnosed with SV over a period of 21 years. The median age of the patients was 81.0 years (range, 24–97 years) and 49 patients (53.3%) were male. Regarding comorbidities, 19 patients (20.7%) had an ASA score higher than III and 51 patients (55.4%) a Charlson index higher than 6. Most patients included in the study presented chronic constipation (84.8%), hypertension (58.7%), dementia (51.1%) or were bedridden (64.1%). The majority of patients complained of abdominal pain (90.2%) and obstipation (97.8%), and all manifested abdominal distension. Regarding symptoms severity, 18.5% of patients presented with colonic gangrene and 9.8% with septic shock.

### Patients characteristics by treatment modality

Table 1 outlines the features of SV patient management. Individuals with conservative management tended to be older, with more severe comorbidities, as revealed by higher Charlson index (OR = 2.088, CI 95% = 1.397–3.125, *p* < 0.001), than cases undergoing urgent or elective surgery. Similarly, conservative management was more frequently used in bedridden patients (OR = 1.550, CI 95% = 1.131–2.123, *p* = 0.008), presenting dementia (OR = 1.835, CI 95% = 1.203–2.801, *p* = 0.004) and chronic constipation (OR = 1.330, CI 95% = 1.116–1.582, *p* < 0.001).

**Table 1:** Sigmoid volvulus patient characteristics according to treatment

	Urgent surgery (***n*** = 27)	Elective surgery (***n*** = 22)	Conservative management (***n*** = 43)	***P*** value
Age (years)	78.0 (24–92)	76.5 (28–96)	85.0 (43–97)	**0.037** ^a^
Sex				
Male	17 (63.0)	13 (59.1)	19 (44.2)	0.254 ^b^
Female	10 (37.0)	9 (40.9)	24 (55.8)	
ASA score				**0.044** ^b^
I	1 (3.7)	0 (0.0)	1 (2.3)	0.673 ^b^
II	1 (3.7)	1 (4.5)	1 (2.3)	0.882 ^b^
III	18 (66.7)	20 (90.9)	30 (69.8)	0.110 ^b^
IV	6 (22.2)	1 (4.5)	2 (4.7)	**0.035** ^b^
V	1 (3.7)	0 (0.0)	9 (20.9)	**0.014** ^b^
ASA score > III	7 (25.9)	1 (4.5)	11 (25.6)	0.101 ^b^
Charlson index	6 (0–11)	5 (0–11)	8 (0–13)	**0.008** ^a^
Charlson index > 6	10 (37.0)	8 (36.4)	33 (76.7)	** < 0.001** ^b^
Comorbid conditions				
Neurological disorder	3 (11.1)	5 (22.7)	11 (25.6)	0.334 ^b^
Hypertension	12 (44.4)	12 (54.5)	30 (69.8)	0.101 ^b^
Myocardial infarction	1 (3.7)	2 (9.1)	2 (4.7)	0.677 ^b^
Chronic heart failure	4 (14.8)	4 (18.2)	13 (30.2)	0.274 ^b^
Diabetes mellitus	6 (22.2)	4 (18.2)	12 (27.9)	0.665 ^b^
Chronic pulmonary disease	6 (22.2)	5 (22.7)	6 (14.0)	0.577 ^b^
Chronic renal failure	4 (14.8)	1 (4.5)	7 (16.3)	0.392 ^b^
Dementia	11 (40.7)	7 (31.8)	29 (67.4)	**0.011** ^b^
Bedridden	14 (51.9)	11 (50.0)	34 (79.1)	**0.020** ^b^
Previous neoplasm	2 (7.4)	3 (13.6)	8 (18.6)	0.423 ^b^
Chronic constipation	21 (77.8)	15 (68.2)	42 (97.7)	**0.004** ^b^
Previous abdominal surgery	5 (18.5)	5 (22.7)	11 (25.6)	0.791 ^b^
Presentation				
Abdominal pain	26 (96.3)	20 (90.9)	37 (86.0)	0.370 ^b^
Obstipation	26 (96.3)	22 (100.0)	42 (97.7)	0.673 ^b^
Abdominal distension	27 (100.0)	22 (100.0)	43 (100.0)	-
Digestive bleeding	0 (0.0)	1 (4.5)	1 (2.3)	0.553 ^b^
Gangrene	10 (37.0)	0 (0.0)	7 (16.3)	**0.004** ^b^
Septic shock	4 (14.8)	0 (0.0)	5 (11.6)	0.190 ^b^
Diagnostic tests				
Abdominal X-ray	27 (100.0)	22 (100.0)	43 (100.0)	-
CT scan	16 (59.3)	11 (50.0)	20 (46.5)	0.579 ^b^

Boldface was used to highlight those significative p-values (lower than 0.05)

Statistics presented as median (min–max) or *n* (%)

*ASA* American Society of Anaesthesiologists

*P* values: ^a^Kruskal-Wallis test, ^b^Pearson’s *χ*^2^ test

### Management outcomes

**Fig. 1:**
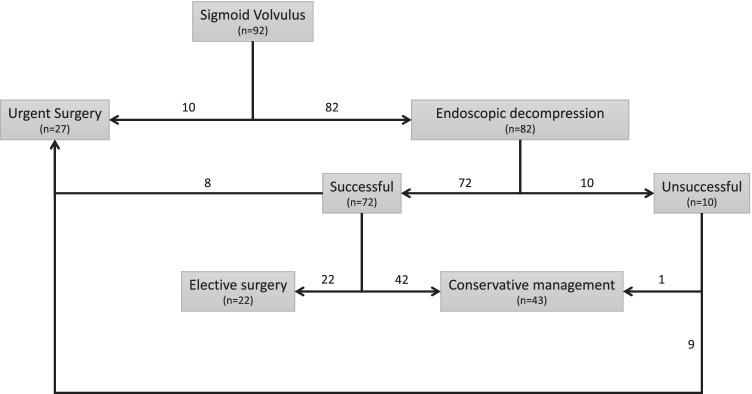
Sigmoid volvulus management flow diagram

 Management outcomes according to SV treatment modality are recorded in Table 2. Conservative management was performed in 43 cases (46.7%), whereas 27 patients (29.4%) underwent emergent surgery and 22 (23.9%) elective surgery. The SV management diagram flow is depicted in Fig. 1. Successful endoscopic decompression was achieved in 72 patients (87.8%), yet volvulus recurred in 34 cases (47.2%). Of the patients successfully treated with endoscopic decompression, 42 (58.3%) underwent conservative management, 22 (30.6%) were planned for elective surgery and 8 (11.1%) presented a recurrence that required urgent surgery. No patients undergoing surgery presented a recurrence. However, at least one further SV episode was recorded in 21 (48.8%) patients with conservative management.

**Table 2:** Outcomes according to sigmoid volvulus management modality

	Urgent surgery (***n*** = 27)	Elective surgery (***n*** = 22)	Conservative management (***n*** = 43)	***P*** value
Endoscopic management				
Endoscopic decompression (yes/no)	8 (29.6)	22 (100.0)	42 (97.7)	**0.003** ^b^
Total decompressions per patient	1 (0–4)	2 (1–5)	1 (0–4)	0.173 ^a^
Surgical procedure				
Resection with anastomosis	4 (14.8)	11 (50.0)	-	**0.024** ^b^
Resection without anastomosis	19 (70.4)	10 (45.5)	-	
Diverting stoma without resection	4 (14.8)	1 (4.5)	-	
Recurrence	0 (0.0)	0 (0.0)	21 (48.8)	-
Reoperation	5 (18.5)	2 (9.1)	-	0.303 ^c^
Length of stay (days)	12 (2–54)	24 (1–52)	5 (1–24)	** < 0.001** ^a^
Readmission	1 (3.7)	4 (18.2)	1 (2.3)	**0.039** ^b^
Any complication in the episode (30 days)	15 (55.6)	11 (50.0)	19 (44.2)	0.647 ^b^
Medical complications in the episode (30 days)	12 (44.4)	6 (27.3)	19 (44.2)	0.365 ^b^
Respiratory complications	10 (37.0)	3 (13.6)	10 (23.3)	0.160 ^b^
Urinary complications	0 (0.0)	1 (4.5)	0 (0.0)	0.200 ^b^
Cardiac complications	1 (3.7)	0 (0.0)	0 (0.0)	0.296 ^b^
Cerebrovascular accident	0 (0.0)	1 (4.5)	1 (2.3)	0.553 ^b^
Septic shock	1 (3.7)	1 (4.5)	6 (14.0)	0.244 ^b^
Postoperative surgical complications (30 days)	9 (33.3)	8 (36.4)	-	0.531 ^c^
Wound disruption	1 (3.7)	0 (0.0)	-	0.551 ^c^
Ileus	3 (11.1)	1 (4.5)	-	0.387 ^c^
Anastomotic leak	2 (7.4)	1 (4.5)	-	0.578 ^c^
Intraperitoneal abscess	2 (7.4)	1 (4.5)	-	0.578 ^c^
Stoma complications	1 (3.7)	0 (0.0)	-	0.551 ^c^
Postoperative bleeding	0 (0.0)	1 (4.5)	-	0.449 ^c^
Surgical site infection	9 (33.3)	6 (27.3)	-	0.444 ^c^
Superficial	4 (14.8)	4 (18.2)	-	0.524 ^c^
Deep	3 (11.1)	1 (4.5)	-	0.387 ^c^
Organ space	4 (14.8)	2 (9.1)	-	0.438 ^c^
Clavien-Dindo				
0	12 (44.4)	11 (50.0)	-	0.475 ^b^
I	0 (0.0)	1 (4.5)	-	
II	2 (7.4)	4 (18.2)	-	
III	2 (7.4)	2 (9.1)	-	
IV	5 (18.5)	2 (9.1)	-	
V	6 (22.2)	2 (9.1)	-	
Clavien-Dindo > 2	13 (48.1)	6 (27.3)	-	0.115 ^c^
Mortality in the episode (30 days)	6 (22.2)	2 (9.1)	16 (37.2)	0.044 ^b^
Cause of mortality (30 days)				
Medical complications	4 (14.8)	1 (4.5)	16 (37.2)	**0.006** ^b^
Surgical complications	2 (7.4)	1 (4.5)	-	0.578 ^c^

Boldface was used to highlight those significative p-values (lower than 0.05)

Statistics presented as median (min–max) or *n* (%)

*P* values: ^a^Kruskal-Wallis test, ^b^Pearson’s *χ*^2^ test, ^c^Fisher’s exact test

The conservative treatment patient group had shorter hospital stay (5 days, range 1–24) than those who underwent urgent (12 days, range 2–54) or elective surgery (24 days, range 1–52) (*p* < 0.001). Regarding complications during the episode (until 30 days after admission), no differences were found between management groups in the likelihood of presenting any complication (*p* = 0.647). Respiratory complications were the most common medical sequelae in all three treatment groups. Analysing postoperative outcomes, urgent and elective surgery reported similar complication rates (33.3%, 36.4% respectively; *p* = 0.531) without differences in Clavien-Dindo classification (*p* = 0.475). Both surgical treatments showed similar anastomotic leak rates (7.4%, 4.5%; *p* = 0.578). Likewise, surgical site infection rates revealed no differences between the two surgery options (*p* = 0.444). Patients with conservative management presented a higher mortality rate (37.2%) than those with urgent or elective surgical management (22.2%, 9.1% respectively; *p* = 0.044). However, no differences in mortality rate were found between urgent or elective surgery (*p* = 0.269).

Patients were stratified following Atamanalp classification and management outcomes are detailed in Table 3. The subgroup of patients with ASA score I–III or those who underwent elective surgery showed lower mortality than those with ASA score IV–V or treated conservatively [10].

**Table 3:** Management outcomes following Atamanalp classification for sigmoid volvulus

Group	Definition	Treatment	Mortality (%)	Morbidity (%)	Recurrence (%)
I A	G 0, A 0, ASA I–III	Endoscopic decompression	16.7	16.7	50.0
		Plus elective surgery	0.0	0.0	0.0
I B	G 0, A I or ASA IV–V	Endoscopic decompression	90.9	90.0	27.3
		Plus elective surgery	0.0	45.5	0.0
II A	G 0, A 0, ASA I–III, E I	Urgent surgery	0.0	33.3	0.0
II B	G 0, A I or ASA IV–V, E I	Urgent surgery	42.9	85.7	0.0
III A	G I, A 0, ASA I–III	Urgent surgery and stoma	0.0	0.0	0.0
III B	G I, A I or ASA IV–V	Urgent surgery and stoma	33.3	83.3	0.0

*A 0*, age < 75 years; *A I*, age ≥ 75 years; *ASA I*, patient with no other disease; *ASA II*, presence of mild systemic disease; *ASA III*, presence of severe systemic disease; *ASA IV*, presence of life-threatening systemic disease; *ASA V*, moribund patient; *E I*, unsuccessful endoscopy; *G 0*, no sigmoid gangrene; *G I*, presence of sigmoid gangrene

**Fig. 2:**
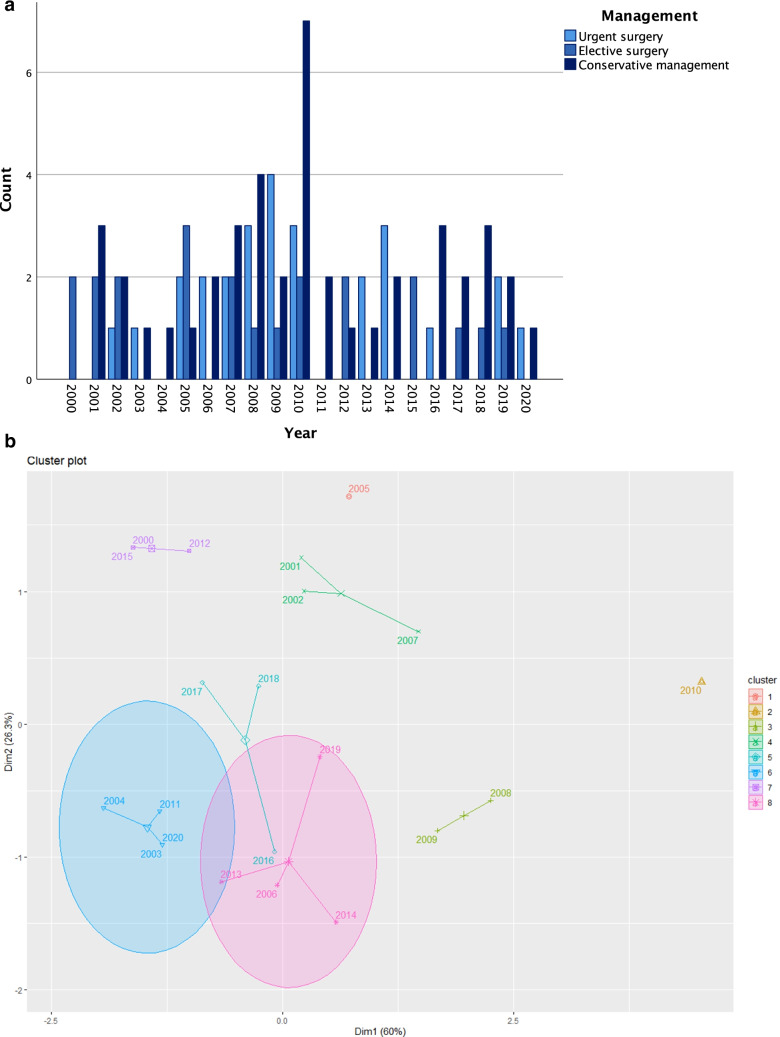
Evolution of the management modalities over the years. **a** Bar chart. **b** Cluster plot

The evolution of the management modalities over the years is depicted in Fig. 2a, but no significant trend was identified. The cluster analysis shaped eight different groups of years. The clusters did not follow a defined temporal pattern, since there were not significant changes in the management during the study period (Fig. 2b).

### Risk factors for morbidity and mortality after treatment of colonic volvulus

Table 4 delineates factors significantly associated with morbidity or mortality in univariable analysis. From these factors, multivariable analysis revealed that only ASA score higher than III was an independent risk factor for presenting any complication (OR = 5.570, 95% CI = 1.740–17.829, *p* < 0.001). However, ASA score > III (OR = 5.574, 95% CI = 2.008–15.477, *p* < 0.001) and chronic heart failure (OR = 2.416, 95% CI = 1.112–5.248, *p* = 0.025) emerged as independent risk factors for medical complications during colonic volvulus episode. In the surgical management patient group, only sigmoid resection with anastomosis was an independent risk factor of surgical complications (OR = 2.824, 95% CI = 1.208–6.600, *p* = 0.022). Analysing mortality in the 30 days after admission, ASA score > III was found to be an independent risk factor (OR = 6.139, 95% CI = 2.629–14.335, *p* < 0.001). Analysing complications in more detail, chronic heart failure (OR = 4.007, 95% CI = 1.939–8.251, *p* < 0.001), chronic pulmonary disease (OR = 4.286, 95% CI = 1.845–9.955, *p* < 0.001) and Charlson index > 6 (OR = 1.636, 95% CI = 1.180–2.270, *p* = 0.015) were independent risk factors for respiratory complications during colonic volvulus episode. Furthermore, presenting colonic gangrene was an independent risk indicator for septic shock (OR = 7.350, 95% CI = 3.883–13.911, *p* < 0.001).

**Table 4:** Univariable and multivariable analysis of factors for morbidity and mortality after management of sigmoid volvulus

	Risk factor	OR	95% CI	***p***-value
Any complication	**ASA score > III**	5.570	(1.740–17.829)	< 0.001
	Colonic gangrene	3.394	(1.196–9.637)	0.015
	Septic shock	8.356	(1.088–64.148)	0.014
Medical complications	**ASA score > III**	5.574	(2.008–15.477)	< 0.001
	Charlson index > 6	1.546	(1.081–2.210)	0.032
	**Chronic heart failure**	2.416	(1.112–5.248)	0.025
	Colonic gangrene	3.568	(1.371–9.284)	0.006
	Septic shock	11.892	(1.551–91.151)	0.003
Surgical complications	**Resection with anastomosis**	2.824	(1.208–6.600)	0.022
Mortality in the episode (30 days	**ASA score > III**	6.139	(2.629–14.335)	< 0.001
	Colonic gangrene	4.048	(1.736–9.436)	0.002
	Septic shock	9.917	(2.211–44.487)	< 0.001
Respiratory complications	**Chronic heart failure**	4.007	(1.939–8.251)	< 0.001
	**Chronic pulmonary disease**	4.286	(1.845–9.955)	< 0.001
	Chronic renal failure	4.200	(1.475–11.956)	0.009
	**Charlson index > 6**	1.636	(1.180–2.270)	0.015
Septic shock	Asa score > III	6.125	(3.410–11.001)	< 0.001
	**Colonic gangrene**	7.350	(3.883–13.911)	< 0.001

Variables in bold were independent factors in the multivariable binary logistic regression model

*ASA* American Society of Anaesthesiologists

### 2-year overall survival analysis

**Fig. 3:**
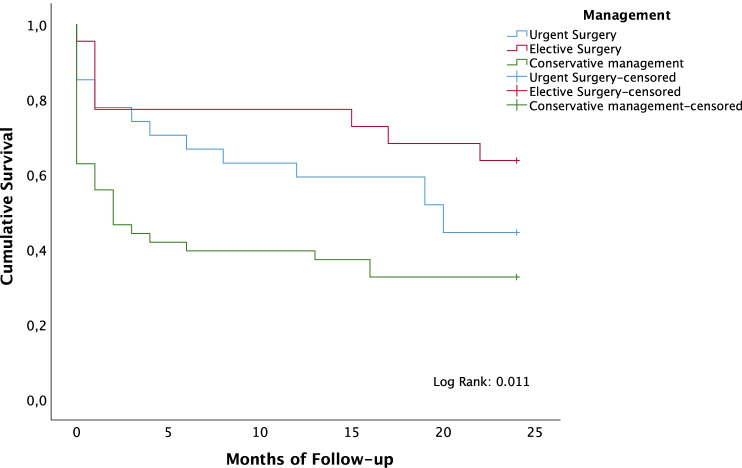
Two-year overall survival curves depending on the management of sigmoid volvulus

**Fig. 4:**
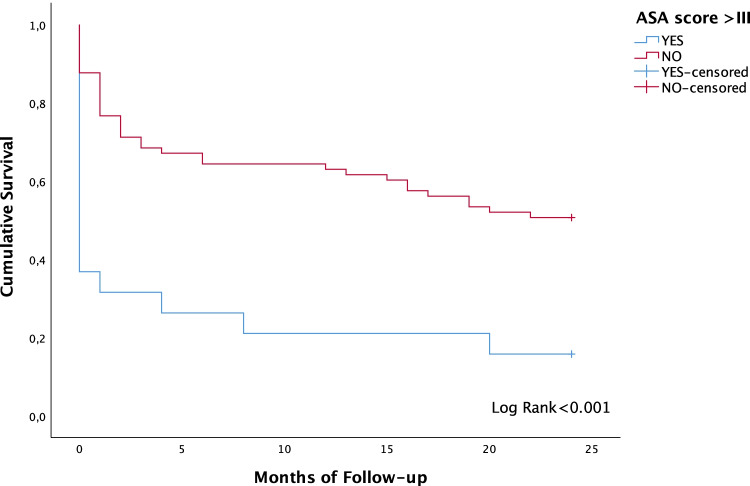
Two-year overall survival curves depending on the ASA score higher than III

In Kaplan–Meier analysis, patients who underwent surgical management showed higher 2-year OS (53.1%; SD = 7.1) than those with conservative management (32.6%; SD = 7.1) (*p* = 0.020). Furthermore, elective surgery revealed a higher 2-year OS (63.6%) than those with conservative management (*p* = 0.011). However, no differences were found between urgent (44.4%; SD = 9.6) and elective surgery (63.6%; SD = 10.3) (*p* = 0.190). A multivariable Cox proportional hazards model was performed to assess possible factors modifying survival. Elective surgical management arose as an independent prognostic factor for higher survival rates (HR = 2.604, 95% CI = 1.185–5.714, *p* = 0.017). Figure 3 illustrates differences in 2-year OS according to management of volvulus. Furthermore, ASA score higher than III was an independent prognostic factor for lower 2-year OS in the multivariable Cox regression model (HR = 0.351, 95% CI = 0.192–0.641, *p* = 0.001). Figure 4 depicts lower 2-year OS for patients with ASA score over III. No differences were found according the management of the SV for the subset of patients with ASA score I–II–III. However, age was an independent prognostic factor for 2-year overall survival in this subgroup. The age cut-point that stratified the patients who could benefit from surgery was 74.8 years, with significant differences in 2-year overall survival (*p* = 0.012). The cohort of patients aged under 74.8 years showed a survival rate of 73.9% (SD = 9.2), while for those with age ≥ 74.8 years, the survival rate was 40.0% (SD = 6.9).

## Discussion

In this retrospective cohort of patients treated for SV at a single institution, several independent risk factors for treatment outcomes have been identified. Survival analysis with assessment of prognostic indicators was also conducted.

In non-endemic countries, colonic volvulus has low incidence and tends to appear in elderly patients with severe morbidities. The median age of patients included in the study was 81.0 years and several comorbid conditions were associated. It is widely reported that SV is commonly identified in frail patients, with neurological disorders, residents of nursing homes or with mental retardation [1, 2, 8].

The first-line therapy in SV management is endoscopic detorsion with flexible sigmoidoscopy, with or without decompression tube placement, followed by elective surgery. However, emergent surgery is mandatory in patients with signs of peritonitis, perforation or unsuccessful endoscopic decompression [4–7]. In the literature, the success rate of endoscopic detorsion of SV ranges from 55 to 95% [6–8, 11–15]. In the present study, endoscopic treatment was successfully achieved in 87.8% of patients. However, recurrence appeared in 47.2% of these, requiring repeated endoscopic decompressions and in 11.1% emergent surgery. Other series reported that at least half of patients treated conservatively could experience recurrence within the first 5 months, with increasing risk of recurrence after each episode up to 75% risk [15–17]. Furthermore, the recurrent SV can reach a mortality rate as high as 40%, so definitive surgical treatment is usually recommended following endoscopic detorsion [1, 2, 4–8, 11–18].

In contrast to recommendations in the literature, in the present study, only 30.6% of SV patients successfully treated endoscopically went on to receive elective surgery. Patients with conservative management tended to be elderly, with severe comorbidities, bedridden, with dementia and chronic constipation, highlighting a reluctance to perform colonic surgery in this frail subset. Identifying predictive factors for complications or mortality could improve candidate selection for elective surgery. Nonetheless, it is worth underlining that patients treated conservatively presented a higher mortality rate than those who underwent surgery. Elective sigmoid colectomy could probably have improved these outcomes [4–6, 17, 18].

Advanced endoscopic therapies were not used as part of SV treatment in our series. Percutaneous endoscopic colostomy and percutaneous endoscopic sigmoidopexy have been suggested as useful management tools for patients not eligible for surgery. Neither technique is exempt from complications, with up to 21% risk of morbidity and 5% risk of mortality reported, so these procedures should generally be reserved for patients deemed to pose a prohibitive degree of risk [4, 6, 19].

Regarding complications during episodes, no differences were found between treatment modalities. Postoperative complications were higher than expected, probably attributable to a high-comorbidity population. Other series have reported complications in up to 60% of cases after surgical treatment of SV [1–4, 20]. Respiratory complications were the most common medical sequelae. Likewise, in other studies, medical morbidity included mainly cardiac and respiratory complications [2, 20, 21]. Concerning surgical complications, ileus (8.2%) and anastomotic leakage (6.1%) were the most frequent complications. Similarly, two studies including 2175 and 2538 patients of colonic volvulus based on the American College of Surgeons National Surgical Quality Improvement Program database found anastomotic leak rates of 4.5% and 6.2%, respectively [3, 22].

Patients with conservative management presented a higher mortality rate (37.2%) than those who underwent urgent or elective surgery (22.2% and 9.1%, respectively) in the 30 days following treatment, probably because of higher age and comorbidity in the conservative group. In line with our outcomes, Kasten et al. demonstrated lower mortality for the surgical resection, notwithstanding the high risk of postoperative morbidity [23]. Other studies revealed high rates of mortality, ranging from 6 to 70%, likely secondary to being a high-risk population [3, 5, 8, 13, 14, 16–18, 22]. In the present study, no differences were found between urgent and elective surgery in terms of complications or mortality rates. However, in other large series, emergent surgery revealed higher morbi-mortality rates than elective surgery [6, 16, 18, 21]. It is conceivable that the small sample size of the patients included in the present study induced a loss of statistical power.

The period of analysis was quite long, so changes in management could emerge. The cluster analysis did not reveal significant changes in the management during the study period. The clustering was probably due to differences in the incidence of SV in our setting.

This study found several risk factors for short-term postoperative outcomes. ASA score higher than III was revealed as a risk factor for presenting any complication. This concurs with a large national analysis by Althans et al., which found that age, SIRS, sepsis, septic shock and ASA class ≥ IV were unmodifiable risk factors common across several complications [3]. ASA score > III and chronic heart failure were risk factors for medical complications. However, the only independent risk factor for surgical complications was sigmoid resection with anastomosis. An expected finding was that ASA score > III was an independent risk factor for mortality during the 30 days after the colonic volvulus event. Previous studies also reported higher mortality rates in elderly patients with severe comorbidities [16, 18]. In a retrospective study on the Nationwide Inpatient Sample conducted by Halabi et al. in the USA, a LASSO algorithm was used to build a predictive model for mortality in colonic volvulus cases, identifying bowel gangrene, peritonitis, coagulopathy, age, use of stoma and chronic kidney disease as strong predictors of mortality [1]. Other authors found age over 60 years, presence of gangrene, peritonitis or shock on admission, recurrent volvulus and conservative management to be risk factors for mortality [4, 14, 16]. Conversely, in a study conducted by Easterday et al., emergent surgery was a predictive factor of postoperative mortality in SV [21]. Atamanalp SS. proposed a noteworthy classification system based on age, ASA grade, disease severity and nature of treatment. The patients of the current series were grouped according Atamanalp classification. Similar to Atamanalp SS. findings, the subset of patients with ASA score I–III or those who underwent elective surgery showed lower mortality than those with ASA score IV–V or treated conservatively, suggesting that patients without morbidities could benefit from elective surgery. This algorithm allows easy patient stratification and rapid selection of the best treatment in each case, providing valuable prognosis information [10].

The present study is one of the few in the literature to analyse survival and prognostic factors in SV. Surgical treatment resulted in higher 2-year OS than conservative management. Similarly, Ifversen et al. used Kaplan–Meier analysis to illustrate mortality, reporting that patients treated with surgery had significantly better survival rates than patients treated conservatively [17]. Regarding prognostic factors for survival after an episode of SV, elective surgery was an independent prognostic factor for higher 2-year OS, whereas ASA score > III emerged as a prognostic factor for a lower 2-year OS. In the subgroup of patients aged over 74.8 years with ASA score I–III, the surgical management proved a benefit over the conservative management. In our institution, SV management decision-making was tailored to each patient, balancing the risks and benefits. Patients usually present severe comorbidities and high anaesthetic risk, so it is important to consider the outcomes of surgery; however, conservative management is associated with higher recurrence and mortality rates. In SV patients initially treated conservatively, therefore, elective surgery could be a better definitive option and conservative management should be reserved only for individuals with truly prohibitive surgical risk. Although there are a small number of patients who could never undergo surgery, surgical treatment after endoscopic detorsion of the SV has probably been an underused option.

The main strengths of the study are our identification of risk factors for complications and survival analysis on a relatively large cohort. Nonetheless, this study has significant limitations, arising from its observational and retrospective design. In the emergency setting, patients with SV were not always managed by a colorectal surgeon, so conservative management could have been selected in high-risk patients. Furthermore, patients treated conservatively were older with more severe comorbidities than those who underwent surgery, implying patient selection bias which could have some influence on further outcomes. The non-randomised nature raises the question of whether the conservative treatment group would have presented better results with surgery, although a randomised study would be very difficult to accomplish considering the low incidence of this condition.

## Conclusion

Successful endoscopic decompression can be achieved in most patients with SV. However, this entails high recurrence, morbidity and mortality rates, so elective surgery is recommended after the first episode. SV patients with severe comorbidities or those who were treated conservatively should be warned about the higher risk of morbidity and even mortality following treatment.

## Data sharing

The data that support the findings of this study are available from the corresponding author upon reasonable request.
